# Supplementary figures and images for: Inferring Regulatory Networks by Combining Perturbation Screens and Steady State Gene Expression Profiles
Source: PLoS One. 2014 Feb 28;9(2):e82393. doi: 10.1371/journal.pone.0082393 (PMC3938831; doi:10.1371/journal.pone.0082393)

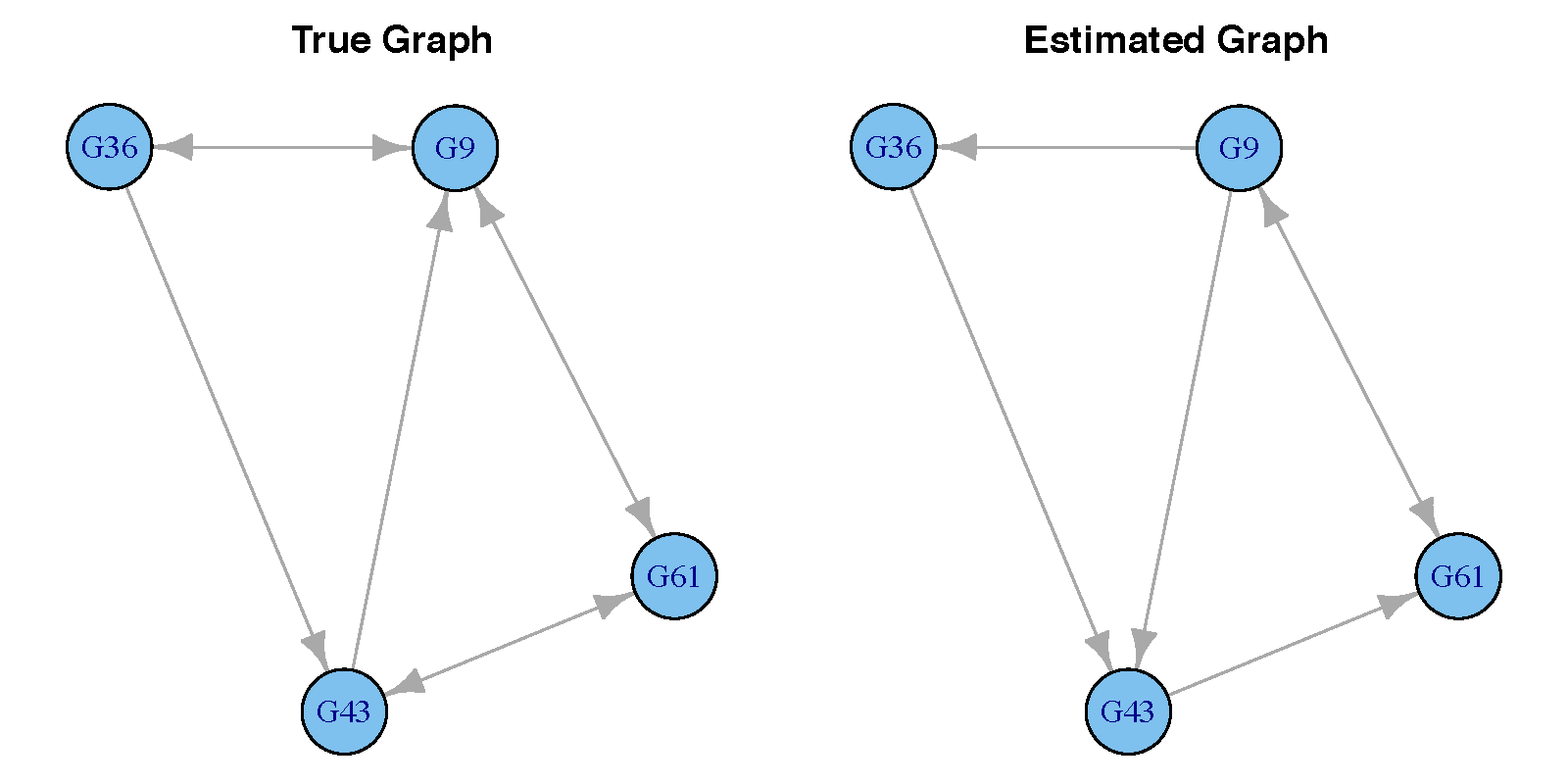

Supplement: Figure S1 — Small cyclic subnetwork example. The true network (left) includes a number of cycles, and the estimate from the RIPE algorithm correctly identifies some of these cycles (right). (TIFF) [file pone.0082393.s001.tif]

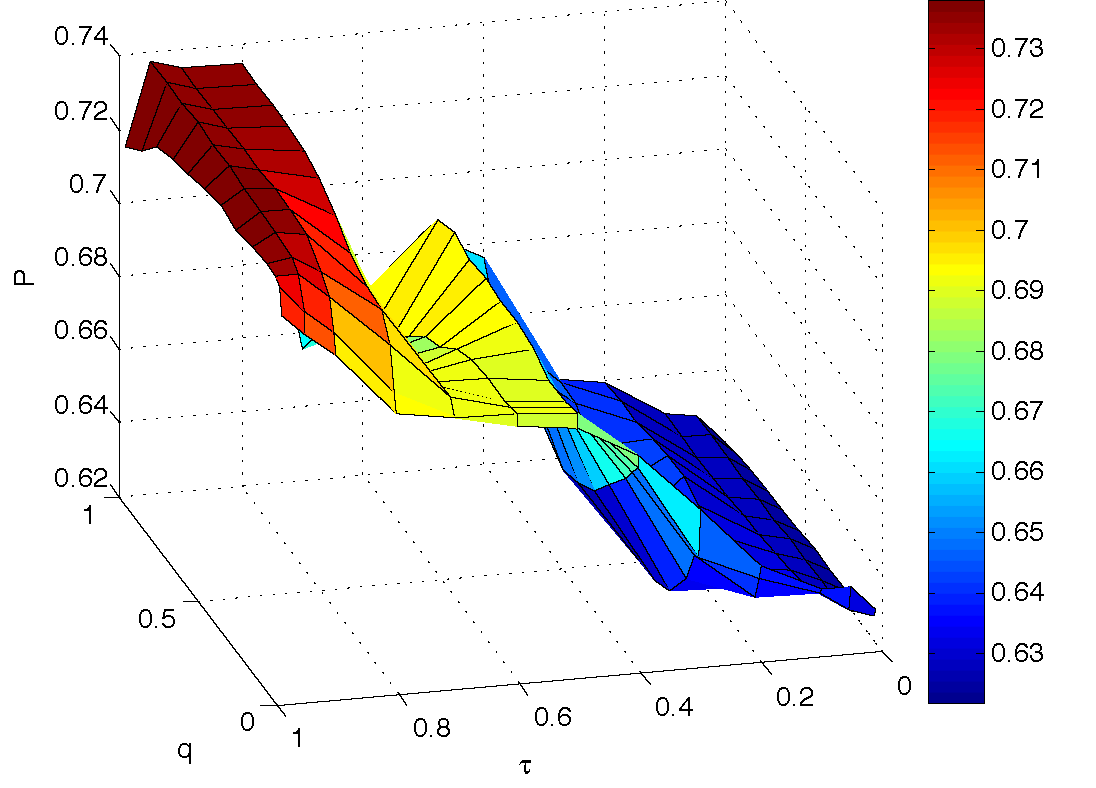

Supplement: Figure S2 — Numerical study on choices of τ and q . Values of the Precision (P) for different combinations of τ (threshold for including an edge in the consensus graph) and q (proportion of highest values of the log-likelihood function used in constructing the consensus graph). (TIFF) [file pone.0082393.s002.tif]

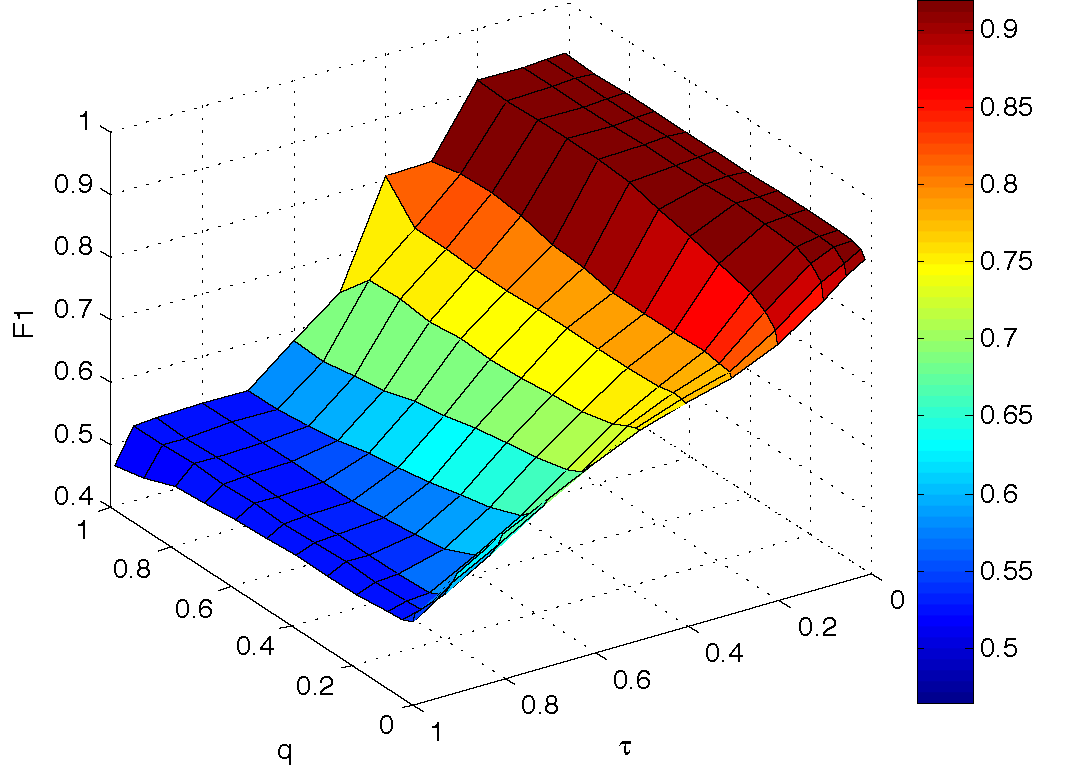

Supplement: Figure S3 — Numerical study on choices of τ and q . Values of the Recall (R) for different combinations of τ (threshold for including an edge in the consensus graph) and q proportion of highest values of the log-likelihood function used in constructing the consensus graph). (TIFF) [file pone.0082393.s003.tif]

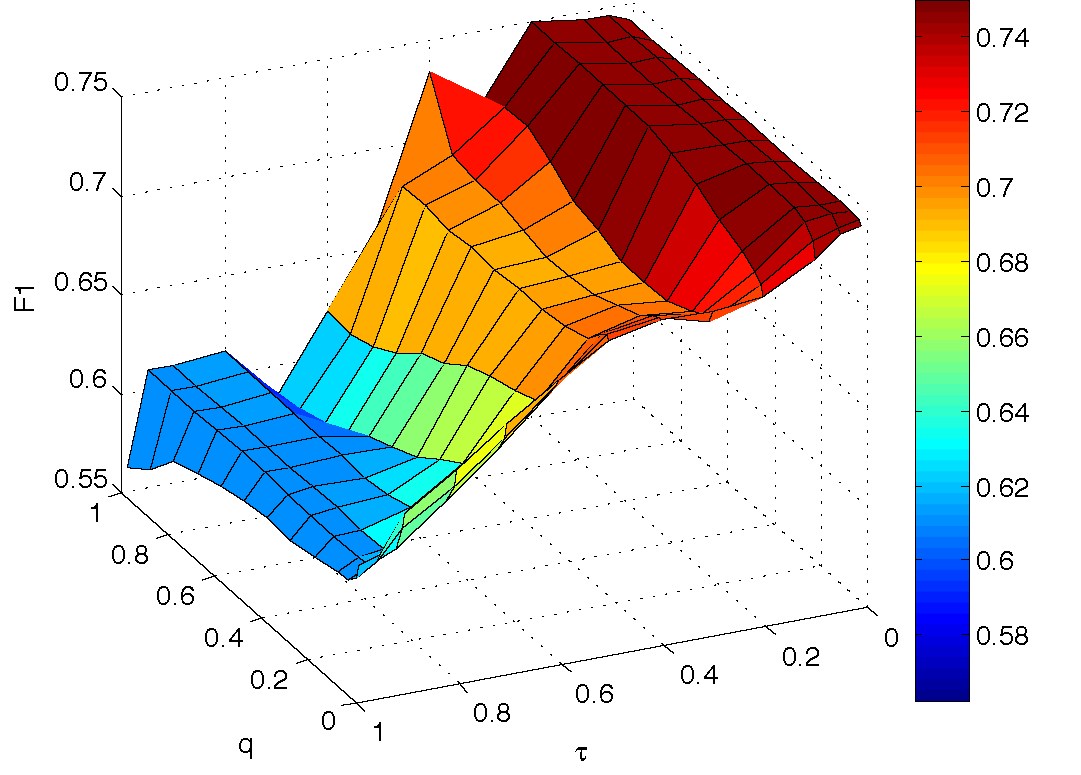

Supplement: Figure S4 — Numerical study on choices of τ and q . Values of the F 1 measure for different combinations of τ (threshold for including an edge in the consensus graph) and q (proportion of highest values of the log-likelihood function used in constructing the consensus graph). (TIFF) [file pone.0082393.s004.tif]

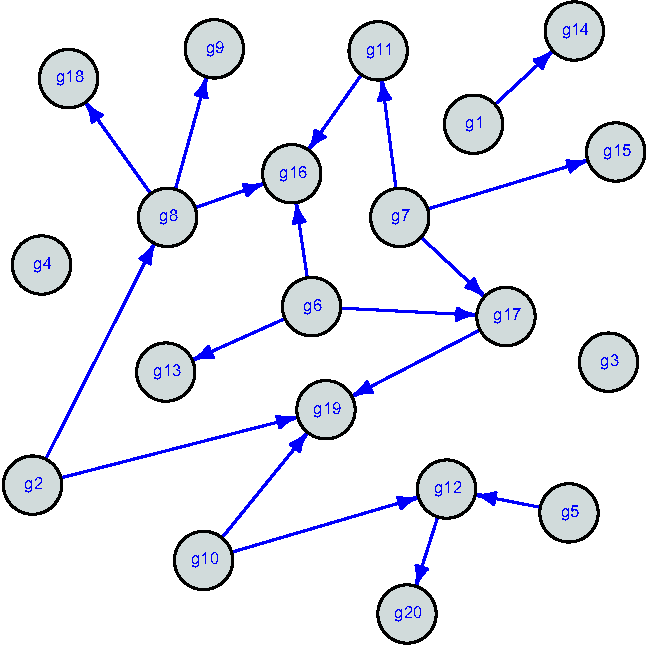

Supplement: Figure S6 — Synthetic regulatory network. (TIFF) [file pone.0082393.s006.tif]

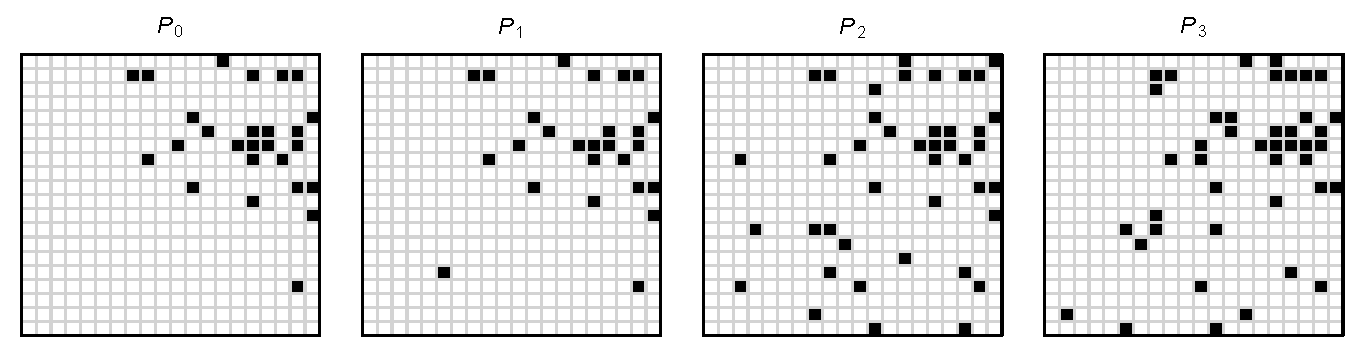

Supplement: Figure S7 — Illustration of influence matrices for synthetic networks. 0: ground truth, 1: 5% of directions reversed, 2: 10% new effects added, 3: 5% directions reversed and 10% new effects added. A black dot in position (i, j) (i.e., in row i and column j) represents that gene i inuences gene j. (TIFF) [file pone.0082393.s007.tif]

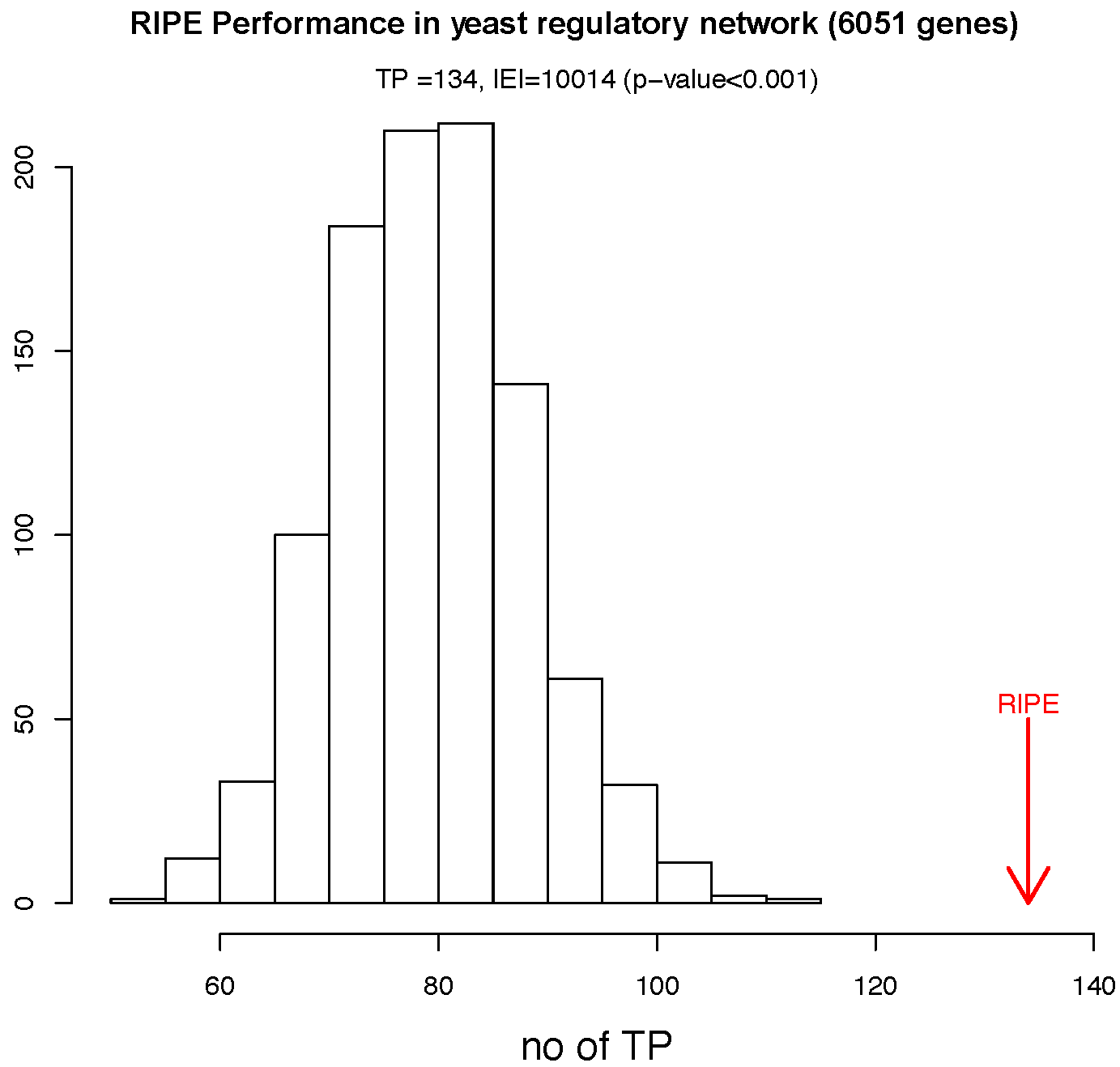

Supplement: Figure S10 — Performance of RIPE in estimating the layered yeast regulatory network. The distribution of number of true positives, in comparison to the number of true positives for the RIPE estimator, for 1000 random graphs with the same number of edges and similar 2-layer structure (p = 6051 genes and k = 269 transcription factors). No random network with equal or larger true positives was observed (p-value<0∶001). (TIFF) [file pone.0082393.s010.tif]

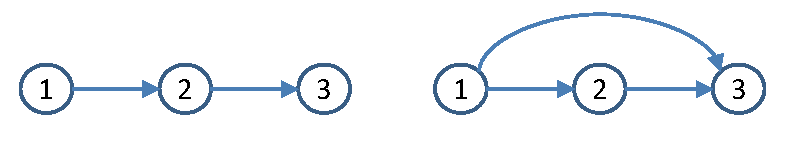

Supplement: Figure S11 — Simple graphs with 3 nodes. (TIFF) [file pone.0082393.s011.tif]
