# Supplementary material for: Inferring Regulatory Networks by Combining Perturbation Screens and Steady State Gene Expression Profiles
Source: PLoS One. 2014 Feb 28;9(2):e82393. doi: 10.1371/journal.pone.0082393 (PMC3938831; doi:10.1371/journal.pone.0082393)
Supplement: Text S1 — Supporting information. (PDF) [file pone.0082393.s012.pdf]

Supporting Information to

Inferring Regulatory Networks by Combining Perturbation Screens and Steady State Gene Expression Profiles

Ali Shojaie[1,#], Alexandra Jauhiainen[2,#], Michael Kallitsis[3,#], George Michailidis[3,*]

[1] **Department of Biostatistics, University of Washington, Seattle, WA, USA**
[2] **Department of Medical Epidemiology and Biostatistics, Karolinska Institutet, Sweden**
[3] **Department of Statistics, University of Michigan, Ann Arbor, MI, USA**

[#] These authors contributed equally to this work.

[*] E-mail: gmichail@umich.edu

Properties of the RIPE Algorithm

Lemma 1. *Suppose that the binary influence matrix $\mathcal{P}$ obtained from perturbation screens correctly reflects the influence of genes in a regulatory network on each other; i.e. all the genes that are influenced by single knocked-out/down genes are correctly included in $\mathcal{P}$. Then,*

(i) $\mathcal{P}$ does not contain sufficient information for estimation of the structure of the underlying regulatory network.

(ii) $\mathcal{P}$ contains sufficient information for estimation of the causal orderings of the nodes of the underlying regulatory network.

Proof. We establish (i) through a counterexample. Consider two simple regulatory networks depicted in Figure S11. It can be seen that these two networks yield the same perturbation data; thus, such data can not provide sufficient information for reconstruction purposes.

To show (ii), first assume G is a DAG. In this case, there exists (at least) one causal ordering of its nodes. (Note: all possible orderings in a DAG are valid causal orderings.) However, by the accuracy of the perturbation data assumed, $\mathcal{P}$ also forms a DAG. In addition, due to the transitivity of the influence matrix, the ordering of the nodes in the influence matrix is the same as that in G. Therefore, a search algorithm for orderings can find a valid causal ordering.

Now, suppose G is not a DAG. The assumed accuracy of the perturbation data implies that the

influence graph represents all the influences of nodes on each other, which in the presence of feedback mechanisms, results in cycles in the influence graph. Denote by $\mathcal{D}$ the DAG of the strong connected components obtained from $\mathcal{P}$. First, note that by construction, and transitivity of the influence graph, the causal ordering of the nodes in $\mathcal{D}$ is a valid partial order of the nodes in $\mathcal{P}$. On the other hand, the orderings among nodes in each strong component (each node in $\mathcal{D}$), are equivalent, and an exhaustive search algorithm (like the backtracking algorithm implemented in RIPE) finds all these equivalent orderings. This implies that the set of orderings obtained by assembling (equivalent) orderings of nodes in all components, together with the partial ordering of nodes in $\mathcal{D}$ gives the list of all orderings consistent with the influence graph. This completes the proof. □

Remark: From the counterexample depicted in Figure S11, it can be seen that including information on the "signs" of the perturbation effects does not help distinguishing the two networks. Further, because of the transitivity of the influences, the result of the lemma continues to hold in the case where all *direct* influences amongst the nodes are accurately included in $\mathcal{P}$ (i.e. without any false positives) and no indirect influences are included. Considering the fact that direct interactions often show most significant knockouts effects, this renders support for more conservative choices of the p-value cutoff.

The following notation is used in the next lemma: $f(n) = O(g(n))$ implies that that there is a positive constant M such that for all sufficiently large values of n, $|f(n)| \leq M\, g(n)$, w hereas $f(n) = o(g(n))$ implies that as $n \to \infty$, $|f(n)/g(n)| < \epsilon$ for any $\epsilon > 0$.

Lemma 2 (Consistency of Estimates)**.** *Assume the data obtained from perturbation screens satisfies the assumptions of Lemma 1 and let s be the total number of true edges in the graph. Suppose that for some* $a > 0$, $p = p(n) = O(n^a)$ *and* $|\mathrm{pa_i}| = \mathrm{O(n^b)}$, *where* $sn^{2b-1}\log n = o(1)$ *as* $n \to \infty$. *Moreover, suppose that there exists* $\nu > 0$ *such that for all* $n \in \mathbb{N}$ *and all* $i \in V$, $\mathrm{Var}\,(X_i|X_{1:i-1}) \geq \nu$ *and there exists* $\delta > 0$ *and some* $\xi > b$ *such that for every* $i \in V$ *and for every* $j \in \mathrm{pa_i}$, $|\pi_{ij}| \geq \delta n^{-(1-\xi)/2}$, *where* π_{ij} *is the partial correlation between* X_i *and* X_j *after removing the effect of the remaining variables.*

Consider the RIPE estimate obtained with an adaptive lasso penalty in the second stage of the algorithm, where the initial weights come from another lasso penalized RIPE step with a tuning parameter satisfying $\lambda^0 = O(\sqrt{\log p/n})$. *Then, if* λ *is chosen so that* $\lambda \asymp dn^{-(1-\zeta)/2}$ *for some* $b < \zeta < \xi$ *a nd* $d > 0$, *we have that with probability converging to 1, the RIPE estimate satisfies the following:*

(i) the edges of the regulatory network are correctly estimated.
(ii) the signs of the regulatory effects (repression/inhibition) are correctly estimated.

Proof. Lemma 1 shows that under the assumptions of this lemma, causal orderings are correctly determined. Then, if the true regulatory network is a DAG, the result follows directly from Theorem 3 of [1]. On the other hand, when the true network consists of cycles, each ordering from the first step of RIPE is a valid ordering. Therefore, for each of the estimated DAGs, claims (i) and (ii) hold by Theorem 3 of [1]. Now, taking $q = 1$ and $\tau = \varepsilon$ for some $\varepsilon > 0$ and small, implies that the

all the estimated edges are included in the graph estimated after model averaging, which in turn implies that (i) and (ii) are satisfied for this network. □

In words, Lemma 2 states that if the perturbation data are accurate, and if adequate samples of steady state data are available (compared to the number of genes in the network, and the number of regulators of each gene), then the RIPE algorithm with the appropriate choices of tuning parameters can correctly estimate both the edges of the network, as well as the sign of the effects (i.e. inductive versus inhibitory effects).

Although this result is asymptotic nature, it nevertheless provides insight into the required number of samples, as long as the available sample size for the steady state data satisfies the requirements of the Lemma related to the total number of edges p, number of regulators of each gene $|\mathrm{pa_i}|$ and the total number of edges in the network s.

The proof mostly builds on proofs establishing consistency of adaptive lasso estimates for estimation problems of directed acyclic graphs with known ordering of nodes [1]. The main difference in case of general regulatory networks is the presence of cycles in the network, where the graph averaging step of the algorithm, coupled with the assumption that the data from perturbation screens is noiseless, gives the desired result. To see this argument from a different point of view, note that, as n increases, DAGs from different orderings corresponding to cycles in the true network have very similar likelihood scores, which results in inclusion of these cycles in the set of final estimates. For instance, consider a bi-directed edge $X_1 \leftrightarrow X_2$. Then DAGs from both orderings have the same likelihoods, and hence the model resulting from averaging the two estimated DAGs will include both edges if an appropriate threshold is used. This is illustrated in Figure S1, where subnetworks corresponding to a small cyclic subgraph from DREAM4-net1 are shown. It is worth noting that the above lemma can be further extended to allow for presence of noise in the perturbation screens; however, this is a rather involved extension and thus beyond the scope of this paper.

References

1. Shojaie A, Michailidis G (2010) Penalized likelihood methods for estimation of sparse high-dimensional directed acyclic graphs. Biometrika 97: 519–538.
